# Salmon nasal cartilage proteoglycan up-regulates *Listeria monocytogenes*-mediated immune response in mice

**DOI:** 10.1016/j.crmicr.2025.100465

**Published:** 2025-08-27

**Authors:** Akio Nakane, Phawinee Subsomwong, Tatsuji Takahashi, Kenichi Ito, Krisana Asano

**Affiliations:** a Department of Biopolymer and Health Science, Hirosaki University Graduate School of. Medicine, Hirosaki, Aomori, Japan; b Department of Microbiology and Immunology, Hirosaki University Graduate School of Medicine, Hirosaki, Aomori, Japan; c Department of Research and Development, Ichimaru Pharcos Co., Ltd., Motosu City, Gifu, Japan

**Keywords:** Proteoglycan, *Listeria monocytogenes*, Host defense, Cytokine, Macrophage, Proteomics

## Abstract

Proteoglycan (PG) is a complex glycohydrate that is widely distributed in the extracellular matrix. We have reported that daily oral administration of PG extracted from salmon (*Oncorhynchus keta*) nasal cartilage modulates the severity and proinflammatory cytokine responses in *Escherichia coli-*stimulated macrophages, and attenuates mouse models of various inflammatory diseases. Thus, PG has shown anti-inflammatory activities. In this study, we investigated the effect of salmon nasal cartilage PG on a bacterial infection by using a mouse model of infection with *Listeria monocytogenes*, which is a facultative intracellular pathogen. PG enhanced production of cytokines including TNF-α, IL-6, IL-12 and IL-10 by heat-killed *L. monocytogenes* in macrophages and dendritic cells. Daily oral administration of PG inhibited proliferation of *L. monocytogenes* in the livers of infected mice. IFN-γ and IL-12 production was enhanced in the PG-treated mice. Uptake of *L. monocytogenes* cells by PG-treated macrophages was decreased, followed reduction of intracellular bacteria growth. Differential proteomic analysis of mouse liver revealed that positive and negative immunological molecules for host defense against *L. monocytogenes* infection were modulated by PG treatment. These results demonstrated that PG can activate immune responses for host defense against *L. monocytogenes* infection through augmentation of cytokines and limiting infection of macrophages. The present study indicates that PG possesses quite different potentials, depending on the host conditions.

## Highlights

• Proteoglycan stimulates *Listeria*-induced cytokine responses in macrophages.
• Proteoglycan treatment enhances host defense to *Listeria* infection in mice.
• *Listeria* uptake is reduced in proteoglycan-treated macrophages.
• Proteoglycan modulates various immunological molecules in mouse liver.

## Graphic abstract

**Figure:**
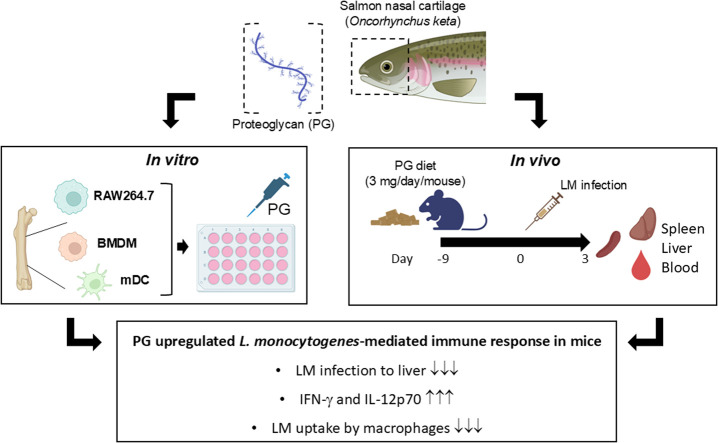

## 1 Introduction

*Listeria monocytogenes* is a facultative intracellular bacterium that is ordinarily non-pathogenic to healthy persons but that it causes opportunistic infections. Debilitated elderly persons and immunocompromised hosts infected by *L. monocytogenes* suffer from septicaemia and meningitis (Gellin and Broome, 1989). Pregnant women, particularly those in their third trimester, often develop chorioamnionitis and their fetuses are infected, leading to septic abortion, stillbirth or birth defects (Vázquez-Boland et al., 2017). Epidemiologic investigations provided evidence that *L. monocytogenes* can be transmitted as an enteric pathogen by contaminated foods, e.g., vegetables, milk, and dairy products (Schlech et al., 1983; Fleming et al., 1985; Linnan et al., 1988), suggesting that natural route of *L. monocytogenes* infection occurs by the oral route. Thereafter these bacteria invade mesenteric lymph nodes via goblet cells of intestinal villa and M cells of Peyers’s patches, and finally form the infectious foci in the spleens and livers (Maudet et al., 2021).

Host defense to *L. monocytogenes* infection depends on macrophages and neutrophils that are activated by tumor necrosis factor (TNF)-α and interferon (IFN)-γ (Huang et al., 1993; Rothe et al., 1993), which are produced by macrophages/dendritic cells and natural killer cells (Serbina et al., 2003; Thäle and Kiderlen 2005), respectively. Interleukin (IL)-12 produced early during infection skews naïve CD4^+^ T cells to T-helper (Th)1 cells and adaptive host defense is performed by Th1-dependent manner using CD4^+^ helper T cells and CD8^+^ killer T cells (Pamer, 2004). Humoral immunity does not appear to play a significant role in clearance of *L. monocytogenes*, although passive immunization with antibodies against virulence factor is protective (Asano at al., 2016). Therefore, *L. monocytogenes* infection has been investigated as a useful model for analysis of cell-mediated immunity.

Proteoglycan (PG) is a complex of glycohydrates consisting of core proteins with one or more covalently attached glycosaminoglycan chains. In cooperation with collagen, fibronectin, and laminin, PG has been shown to be involved in cellular proliferation and adhesion (Danen and Yamada, 2001). Structural information on PG extracted from salmon (*Oncorhynchus keta*) nasal cartilage has been reported (Kakizaki et al., 2011, ID amino acid number BAJ61837.1). Based on the deduced amino acid sequence, it was identified as an aggrecan lacking the keratin sulfate domain, which is commonly present in mammalian aggrecan. We have previously demonstrated that salmon (*O. keta*) nasal cartilage-derived PG can suppress inflammatory responses (Sashinami et al., 2006). In addition, daily oral administration of PG attenuates the severity of experimental inflammatory colitis (Mitsui et al., 2010), autoimmune encephalomyelitis (Sashinami et al., 2012), and collagen-induced arthritis (Yoshimura et al., 2014), and type I allergy (Ono et al., 2018).

In this study, we investigated the effect of PG on immune responses to *L. monocytogenes*, a Gram-positive bacterium, *in vitro* and *in vivo*. Different from previous findings, it was demonstrated that PG augmented proinflammatory cytokine responses and host defense to *L. monocytogenes* infection.

## 2 Materials and methods

### 2.1 Preparation of PG

Heads of salmon (*Oncorhynchus keta*) were purchased from Fisheries companies in Aomori Prefecture, Japan. PG was extracted from salmon (*O. keta*) nasal cartilage slices, as previously reported by Majima et al. (2001). Briefly, frozen salmon nasal slices were dissolved in a solution of 4 % acetic acid to extract PG. PG extract was lyophilized and used for the present experiments. We used high-performance liquid chromatography analyses (TSKgel G5000PWXL column, Tosoh Corporation, Tokyo, Japan) and differential refractive index detection by RID-10A (Shimadzu, Kyoto, Japan) for the quantitative and qualitative analysis of PG. The PG purity was >99 % (Tomonaga et al., 2017). Purified PG (105 g, lyophilized dry weight) was obtained from 15 kg of the raw material (wet weight). This processing was carried out at Ichimaru Pharcos Co., Ltd., Motosu, Gifu, Japan.

### 2.2 Mice

Six-week-old C57BL/6JJcl female mice were purchased from Clea Japan, Inc., Tokyo, Japan. Mice were maintained under specific pathogen-free conditions, and in a temperature-controlled room (22 °C) on a 12-h light-dark cycle at the Institute for Animal Experimentation, Hirosaki University Graduate School of Medicine. Food and water were given ad libitum. Mice were used for experiments at 8-week-old (body weight ca. 23 g). All animal experiments were carried out in accordance with the institutional for Animal Care and use Committees (IACUC)/ethics committees of Hirosaki University.

### 2.3 Mouse macrophages including RAW264.7 cells and bone marrow-derived macrophages (BMDM), and bone marrow-derived myeloid dendritic cells (mDC)

Mouse macrophage cell line RAW264.7 cells (Sumitomo Pharma, Osaka, Japan) were cultured at 37 °C under 5 % CO_2_ in Dulbecco’s modified Eagle medium (DMEM; Nissui Pharmaceutical Co., Tokyo, Japan), supplemented with 10 % fetal bovine serum (FBS; JRH Bioscience, Lenexa, KS), 0.03 % l-glutamine (Wako Pure Chemical Industries, Osaka, Japan), and 1 × Antibiotic-Antimycotec (Gibco; Thermo Fisher, Waltham, MA).

Preparation of BMDM and mDC was carried out as reported previously (Teng et al., 2024; Madaan et al., 2014). Briefly, femurs and tibias were collected from C57BL/6J mice. After removal of the epiphyses, the bone marrow cells were flushed out with RPMI 1640 medium (Nissui) and filtered through a 100-μm stainless steel mesh. The cells were harvested by centrifugation at 200 × *g*, 4 °C for 5 min, and resuspended in 0.85 % NH_4_Cl. Following incubation for 5 min, the cells were collected by centrifugation and washed twice with RPMI 1640 medium. The cells were then seeded into a 75 cm^2^ cell culture flask and cultured at 37 °C, 5 % CO_2_ in RPMI 1640 medium supplemented with 20 ng/mL macrophage colony-stimulating factor (M-CSF; Wako) for BMDM or with 100 ng/mL granulocyte-macrophage colony-stimulating factor (GM-CSF; Wako) for mDC. On Day 4, a half volume of the culture supernatant was replaced with fresh RPMI 1640 medium containing 10 ng/mL M-CSF for BMDM or 100 ng/mL GM-CSF for mDC. On Day 8, the adherent cells were harvested and counted for further use.

### 2.4 Administration of PG

Most experiments were done using 3 mg/day/mouse. Briefly, PG was dissolved in sterilized distilled water (DW) at a concentration of 15 mg/mL. The PG solution was then mixed with powdered diet (CE-2; Clea Japan Inc.) at a final concentration of 0.75 mg/g. In control mice, DW was mixed with powdered diet. Food and water were provided ad libitum. Mice consumed approximately 4 g of diet daily. Daily intake of diet was calculated based on the reduction in diet weight over 24 h for each mouse (*n* = 15 from three experiments). Therefore, daily ingestion of PG was estimated to be 3 mg per mouse. Intake of PG was continued from 9 days before infection to the day of sampling. Likewise, in experiments on dose response of PG, daily ingestion of PG at 1 mg, 3 mg, or 5 mg per mouse was continued from 9 days before infection to the day of sampling.

### 2.5 *L. monocytogenes infection and preparation of heat-killed L.* monocytogenes *(HKLM)*

*L. monocytogenes* 1b 1684 was grown in tryptic soy broth (TSB; BD Diagnosis Systems, Sparks, MD) for 16 h at 37 °C. The concentration of the bacterial cells was adjusted with phosphate-buffered saline (PBS) spectrophotometrically at 550 nm. Mice were infected intraperitoneally with 0.2 mL of solution containing 1 × 10^6^ colony-forming units (CFU) of viable *L. monocytogenes* cells in PBS. HKLM was obtained by heating using Dry ThermoUnit DTU-28 (TAITEC Co., Koshigaya, Saitama, Japan) at 95 °C for 45 min. All organisms were killed by this treatment. HKLM resuspended in PBS at a concentration of 1 × 10^9^ cells/mL was stored at −80 °C until use.

### 2.6 Determination of the number of viable *L. monocytogenes* cells in liver

The livers were aseptically removed from mice infected 72 h after *L. monocytogenes* infection. The resected liver was weighed and 1 g (wet weight) was extracted. The extracted liver specimens were homogenized in PBS using Glass tissue grinder (ACG Techno Glass Co. Ltd., Kawashiri, Shizuoka, Japan). The number of bacteria in the liver specimen was counted by plating 10-fold dilutions of organ homogenates on tryptic soy agar (TSA; BD Diagnosis Systems). Colonies were routinely counted 24 h later. The number of colonies was converted to the whole liver weight and expressed as CFU/organ.

### 2.7 Spleen cell cultures

For preparation of spleen cell cultures, spleen was collected and squeezed in RPMI 1640 medium. Spleen cells were filtrated through stainless mesh (size, 100 μm). Then, the erythrocytes were lysed with 0.85 % NH_4_Cl. After washing three times with RPMI 1640 medium, the spleen cells were resuspended in RPMI 1640 medium supplemented with 10 % FBS, 0.03 % l-glutamine, 2-mercaptoethanol (Wako) and 1 × Antibiotic-Antimycotec and maintained at 37 °C under 5 % CO_2_. For investigation, spleen cells were obtained from mice infected 4 days before. Naive mice were used as control. Spleen cells were seeded on 24-well culture plates at 1 × 10^7^ cell/well and then added 4 × 10^7^ cells of HKLM to each well 24 h later. Culture supernatant fluids were harvested after incubation 37 °C under 5 % CO_2_ for 24 h and proceeded to cytokine assays.

### 2.8 Cytokine assay

The titres of TNF-α, IL-6, IL-10 and IL-12p70 were measured using mouse ELISA kits purchased from Invitrogen, Carlsbad, CA. The protocol followed the manufacturer’s instructions.

### 2.9 Bacterial infection assay

Bacterial infection assay was carried out as reported by our previous studies ( Subsomwong et al., 2024). Briefly, RAW 264.7 cells (1 × 10^6^ cells/mL/well) were seeded in 24-well plates and incubated at 37 °C, 5 % CO_2_ for 24 h, and then treated with PG (250 μg/mL) and/or HKLM (4 × 10^7^ cells/mL) for 24 h. Then, RAW 264.7 cells were washed with washing medium (DMEM without FBS and Antibiotic-Antimycotic). Meanwhile, *L. monocytogenes* was grown in TSB for 16 h at 37 °C, washed with PBS twice, and resuspended in antibitoic-free DMEM. RAW 264.7 cells were infected with *L. monocytogenes* at multiplicity of infection (MOI) = 100. At 60 min of infection, the uninfected bacterial cells in the supernatant were removed. The cells were then washed twice with washing medium and once with PBS. Non-invasive bacterial cells were eliminated by supplementing the culture medium with 120 μg/mL of gentamicin (Wako). At 1 h and 4 of incubation, the cells were washed twice with washing medium and once with PBS. The intracellular bacterial cells were enumerated by lysing cells with 1 % 3-[(3-Cholamidopropyl)-dimethylammonio]−1-propanesulfonate (DOJINDO, Kumamoto, Japan) for 15 min. The number of bacteria was evaluated by plate count assay on TSA after 24 h incubation at 37 °C.

### 2.10 Uptake of *L. monocytogenes* by RAW 264.7 cells

The bacterial uptake experiment was done according to our previous paper (Subsomwong et al., 2024). Briefly, RAW 264.7 cells (1 × 10^5^ cells/well) were seeded in a 4-Chamber 35 mm glass bottom dish (AGC Techno Glass Co, LTD., Tokyo, Japan) and incubated at 37 °C, 5 % CO_2_ for 24 h. Pretreatment with PG and/or HKLM and cultivation of *L. monocytogenes* for infection was carried out as described in *Bacterial infection assay*. The *L. monocytogenes* cells were collected, washed twice with PBS and adjusted to optical density (OD) 600 nm = 1.0 in PBS. The bacterial cells were stained with Vybrant™ DiO Cell-Labeling Solution (Invitrogen, Eugene, OR) as described in the manufacturing instructions. Briefly, 5 μL of DiO dye was added to 1 mL of *L. monocytogenes* and incubated at 37 °C for 30 min under dark condition. The excess dye was removed by washing twice with PBS and the labeled bacterial cells were resuspended in antibiotic-free DMEM. RAW 264.7 cells were washed with washing buffer and infected with DiO-stained *L. monocytogenes* at MOI = 10. At 90 min of infection, the bacterial cells were removed, and the cells were washed twice with washing medium and once with PBS. Non-invasive *L. monocytogenes* was eliminated by 120 μg/mL of gentamicin for 1 h at 37 °C, 5 % CO_2_. Then, the cells were washed in PBS and the Dio-stained *L. monocytogenes* was observed under a fluorescence microscope. The cells infected with *L. monocytogenes* were randomly taken pictures from five different areas of the dish. The number of infected cells with intracellular green fluorescence was calculated by using the provided software (Hybrid Cell Count) by fluorescence microscope. The percentage of infected cells was calculated by the following equation. [ % *L. monocytogenes* uptake cells = (Number of infected cells/ Number of total cells) x 100]. In this study, the percentage of cells taken up *L. monocytogenes* was obtained from 4 independent experiments, and the value was the average of five areas in each group.

### 2.11 Differential proteomic analysis of liver from PG-treated mice

Liver samples from mice administered with PG for 9 days or untreated control mice (*n* = 6 per group) were homogenized in PBS. The proteins were precipitated using acetone. Briefly, the supernatant from the homogenized liver was transferred into two-milliliter microcentrifuge tubes, and four times the sample volume of cold (−20 °C) acetone was added. After the vortex, the tubes were incubated overnight at −20 °C. After centrifugation at 14,000 x *g* for 10 min at 4 °C, then the remaining acetone in open tubes was allowed to evaporate at room temperature for 30 min. The protein pellet was dissolved in PBS, and protein concentration was measured using Bradford protein assay (Bio-Rad Laboratories, Richmond, CA). Proteins were first denatured using 50 % trifluoroethanol and reduced with 4 mM dithiothreitol. Alkylation of free cysteine residues was performed before trypsin digestion, and the desalted peptides were subsequently separated through liquid chromatography. A mass spectrometer was used to analyze the samples. The resulting spectra were compared against *Mus musculus* proteins database using ProteinPilot software (v5.0.1, AB Sciex) and the resulting file was imported to PeakView (v2.2.0, AB Sciex). The peak from SWATH-MS runs were extracted with a false discovery rate of < 1 %. The peak area of each protein was normalized to the total peak areas using the MarkerView software program (v1.3.0.1, AB Sciex). The differences in protein expression between PG-treated mice and non-treated controls (NC) were determined by the relative abundance of proteins in each group. The cut-off values of -log10(P-value) > 1.3 and log2FC > 0 or log2FC < 0 were used for the analysis of DEPs between the PG-treated and NC groups. The interaction network generated by STRING was visualized in Cytoscape software (http://cytoscape.org/;v.3.10.1). The Cluego plug-in of Cytoscape software was designed for the visualization and functional analysis of gene ontology (GO) terms, and GO annotates genes to immune system terms.

### 2.12 Statistical analysis

**Fig. 1:**
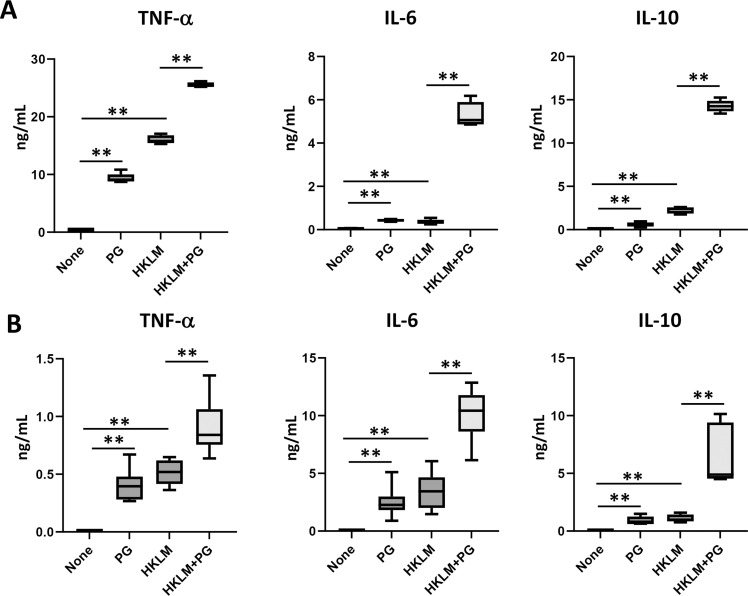
Effect of PG on HKLM-induced cytokine production in mouse macrophages. RAW 264.7 cells (A) or BMDM (B) were seeded in a 24-well plate at 2 × 10^6^ cells/mL/well and cultured for 48 h with 4 × 10^7^ cells of HKLM in the presence or absence of 250 μg/mL of PG. The levels of TNF-α, IL-6 and IL-10 in the culture supernatant were determined by ELISA kits (*n* = 6 from 2 independent experiments). *P-*value was calculated using the Unpaired *t*-test (**P < 0.01).

**Fig. 2:**
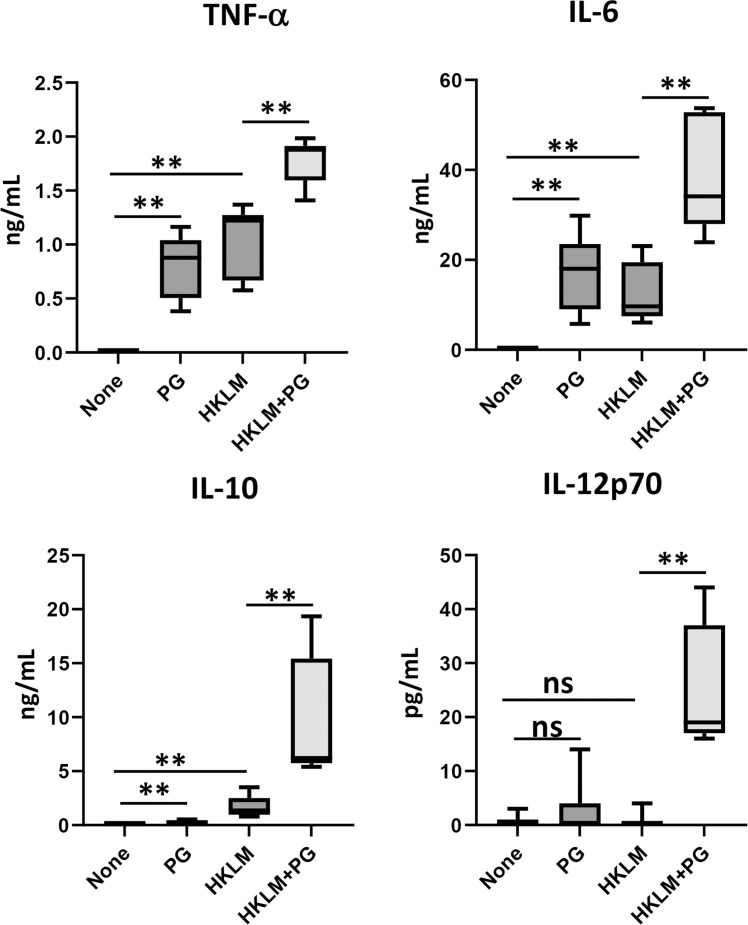
Effect of PG on HKLM-induced cytokine production in mouse dendritic cells. mDC were seeded in a 24-well plate at 2 × 10^6^ cells/mL/well and cultured for 48 h with 4 × 10^7^ cells of HKLM in the presence or absence of 250 μg/mL of PG. The levels of TNF-α, IL-6, IL-10 and IL-12p70 in the culture supernatant were determined by ELISA kits (*n* = 6 from 2 independent experiments). *P-*value was calculated using the Unpaired *t*-test (**P < 0.01, ns=not significant).

**Fig. 3:**
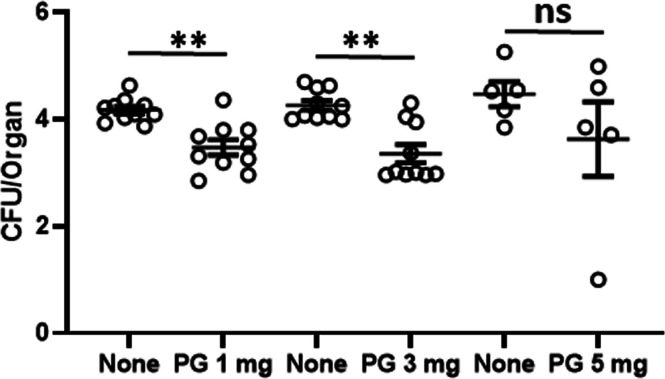
Effect of PG administration on host defense to *L. monocytogenes* infection in the phase of innate immunity of mice. C57BL/6 mice were fed with various doses of PG-supplemented diet (1 mg/day, 3 mg/day and 5 mg/day, respectively) from 9 days before infection to sampling. Control mice were fed normal diet. Then, these mice were infected intraperitoneally with 1 × 10^6^ CFU of *L. monocytogenes*. The numbers of bacteria in the livers of mice were determined at 72 h after infection. Each data represents the mean ± SEM (error bar) for a group of 10 mice from two experiments. *P-*value was calculated using the Mann-Whitney *U test* (*P < 0.05 and **P < 0.01, ns = not significant).

**Fig. 4:**
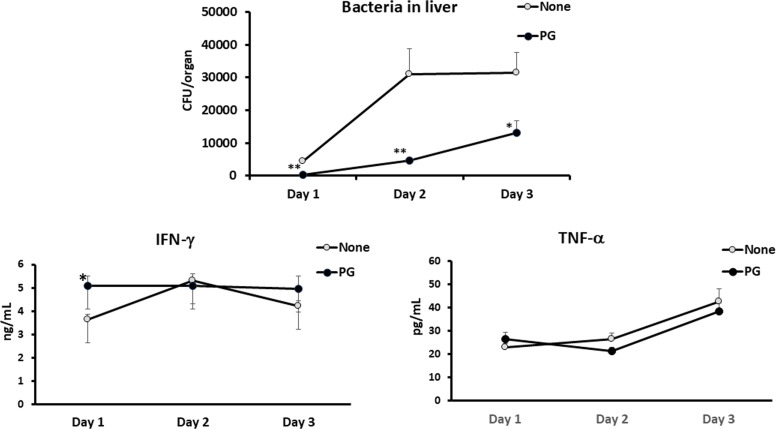
Effect of PG administration on bacterial growth in the organs and cytokine production in the bloodstream of *L. monocytogenes*-infected mice. C57BL/6 mice were fed with PG-supplemented diet (3 mg/day) from 9 days before infection to sampling. Control mice were fed normal diet. Then, these mice were infected intraperitoneally with 1 × 10^6^ CFU of *L. monocytogenes*. The numbers of bacteria in the livers of mice were determined on Day 1, Day 2 and Day 3 after infection (A). In parallel, titres of IFN-γ and TNF-α in the bloodstream were determined (B). Each data represents the mean ± SEM (error bar) for a group of 8 mice from two experiments. *P-*value was calculated using the Mann-Whitney *U test* in bacterial number and cytokine titres in the Unpaired *t*-test (*P < 0.05 and **P < 0.01).

**Fig. 5:**
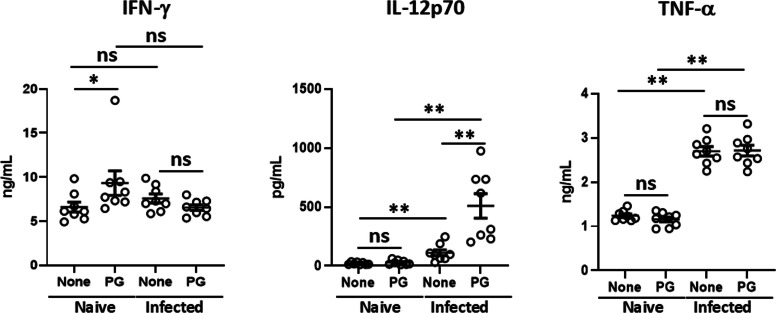
Effect of PG administration on cytokine production in the spleen cell cultures obtained from mice infected with *L. monocytogenes*. C57BL/6 mice were fed with PG-supplemented diet (3 mg/day) from 9 days before infection to sampling. Control mice were fed normal diet. Then, these mice were infected intraperitoneally with 1 × 10^6^ CFU of *L. monocytogenes*. Spleen cells were prepared from mice at 96 h after infection and non-infected mice, respectively. They were cultured with HKLM for 24 h. The levels of cytokines in the culture supernatants were quantitated. Each data represents the mean ± SEM (error bar) for a group of 8 mice from two experiments. *P-*value was calculated using the Unpaired *t*-test (*P < 0.05 and **P < 0.01, ns = not significant).

**Fig. 6:**
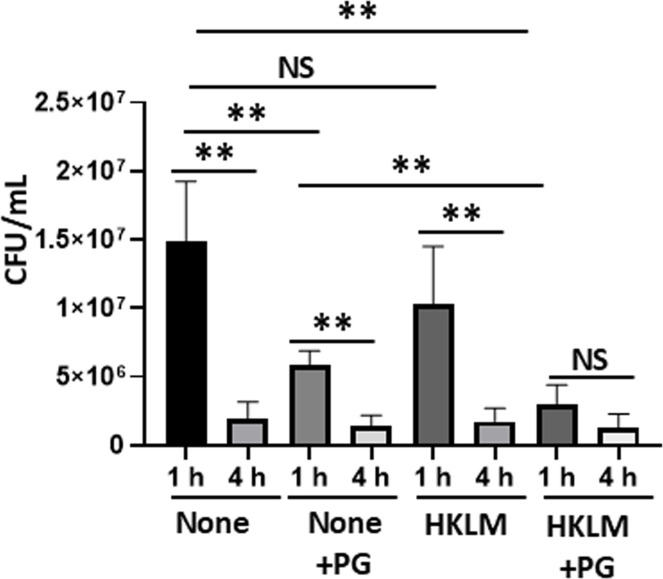
Effect of PG and HKLM on intracellular *L. monocytogenes* in mouse macrophages. RAW 264.7 cells were seeded in a 24-well plate at 1 × 10^6^ cells/mL/well and cultured for 48 h with 4 × 10^7^ cells of HKLM in the presence or absence of 250 μg/mL of PG. The intracellular bacterial number was then evaluated by plate count assay at 1 h and 4 h of infection (*n* = 6 from 2 independent experiments). Each data represents the mean ± SEM (error bar). *P-*value was calculated using the Mann-Whitney *U test* (*P < 0.05 and **P < 0.01, ns = not significant).

**Fig. 7:**
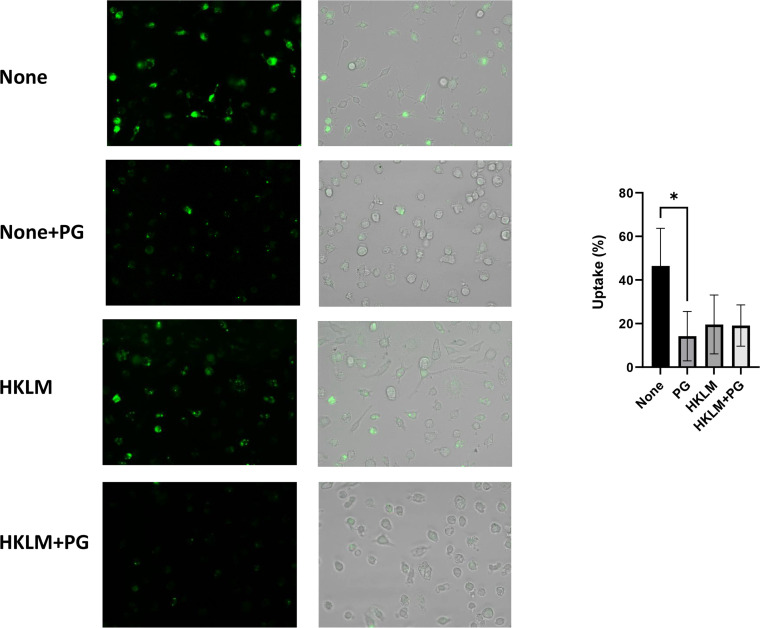
Effect of PG and HKLM on uptake of *L. monocytogenes* by mouse macrophages. (A) Fluorescence microscopy. RAW 264.7 cells were seeded in a 4-chamber glass bottom dish at 1 × 10^5^ cells/mL/well and cultured for 24 h with 4 × 10^6^ cells of HKLM in the presence or absence of 250 μg/mL of PG. *L. monocytogenes* was stained using Vybrant™ DiO Cell-Labeling Solution before cell infection. The confluent RAW 264.7 cells were infected with *L. monocytogenes* at MOI = 10 for 1 h. Extracellular bacterial cells were eliminated by treating the cells with gentamicin for additional 1 h. The cells were washed, and the fluorescence signal was observed under fluorescence microscope. The average of percentage of uptake cells were calculated from five areas (*n* = 20 from 4 independent experiments). (B) Uptake of *L. monocytogenes* ( %). *P* value was calculated using Kruskall Wallis H-test (*P < 0.05).

The data were analyzed using GraphPad Prism (Version 9.5.1, GraphPad Software, San Diego, CA). Standard deviation (SD) analyses were carried out in Figs. 1, 2, 7 and standard error of the mean (SEM) analyses were used in Fig. 3, Fig. 4, Fig. 5, Fig. 6. A *P*-value of <0.05 was considered statistically significant.

### 2.13 Data availability

The proteomic data of liver samples from mice administered PG and untreated control have been deposited in Proteome Xchange and jPost Repository under accession numbers PXD061495 and JPST003673.

## 3 Results

### 3.1 Effect of PG on cytokine production induced by HKLM in macrophages and mDC

The effect of PG on cytokine production induced by HKLM in macrophages including RAW264.7 cells (Fig. 1A) and BMDM (Fig. 1B) was investigated. IL-6 and IL-10 production induced by HKLM or PG was low, but their titres were synergistically enhanced in the presence of HKLM and PG. In contrast, the effect of PG might be additive on TNF-α production because PG alone induced TNF-α production. IL-12p70 was not detected in RAW264.7 cells or BMDM even in addition of both HKLM and PG (data not shown). Next, the effect of cytokine production induced by HKLM was investigated in mDC (Fig. 2). IL-10 production was synergistically enhanced by HKLM and PG, while the titres of TNF-α and IL-6 were additively increased. On the other hand, IL-12p70 was significantly detected by stimulation with both HKLM and PG, although the titres of IL-12p70 were not increased by HKLM or PG alone compared with those in the non-stimulated cultures.

### 3.2 Effect of PG treatment on host defense to *L. monocytogenes* infection in mice

From 9 days before infection, mice were fed daily with different doses of PG-supplemented diet or control diet. Mice of both groups were infected intraperitoneally with *L. monocytogenes* and the number of *L. monocytogenes* in the livers was determined 3 days later (Fig. 3). Elimination of bacterial cells from the livers was enhanced, especially when 1 mg/day/head or 3 mg/day/head of PG was administered (*P* < 0.01). From these results, the following experiments were carried out using mice treated with 3 mg/day/head of PG.

### 3.3 Effect of PG treatment on serum cytokines during *L. monocytogenes* infection

We investigated the effect of PG treatment on production of TNF-α and IFN-γ, which are essential for host defense against *L. monocytogenes* infection. After infection with *L. monocytogenes*, both cytokines in the bloodstream were monitored at 24 h, 48 h and 72 h (Fig. 4). The IFN-γ titres of PG-treated mice at 24 h of infection were significantly higher than those of non-treated mice (*P* < 0.05). Thereafter, IFN-γ titres at 48 h and 72 h were comparable between PG-treated mice and control mice, although bacterial numbers in the livers were markedly reduced. On the other hand, TNF-α titres were not different between two groups at any times examined.

### 3.4 Effect of PG treatment on cytokine response in spleen cell culture

We further investigated the effect of PG treatment on production of TNF-α and IFN-γ, and also IL-12p70, which is an essential cytokine for differentiation of naïve T cells to Th1 cells, in the spleen cell cultures. Spleen cells from naïve or *L. monocytogenes*-infected mice with or without PG treatment were stimulated with HKLM, and cytokines in the culture supernatant fluids were estimated (Fig. 5). IFN-γ production was significantly enhanced in PG-treated group compared with that in control group in naïve mice (*P* < 0.05), whereas the effect of PG treatment on IFN-γ production was not observed in the infected mice. IL-12p70 production was not affected by PG treatment in naive mice. However, the infected mice showed enhancement of IL-12p70 production by PG treatment (*P* < 0.01). Moreover, IL-12p70 response was highly augmented in the PG-treated and infected mice compared with that in the PG-treated naïve mice (*P* < 0.01). On the other hand, TNF-α production was not affected by PG treatment in either naïve or infected mice, although *L. monocytogenes* infection up-regulated TNF-α production in PG-treated mice and non-treated control mice (*P* < 0.01).

### 3.5 Effect of PG on bactericidal activity of macrophages

It is known that activated macrophages phagocytose and kill intracellular *L. monocytogenes*, although resident macrophages are a suitable habitat (Pizarro-Cerdá, 2018). Therefore, we investigated the bactericidal activity of PG-pretreated macrophages to assess whether PG can activate macrophages. RAW264.7 cells were pretreated with HKLM in the presence or absence of PG for 24 h and the number of intracellular *L. monocytogenes* cells at 1 h and 4 h after exposure to bacteria (Fig. 6). Surprisingly, the number of intracellular *L. monocytogenes* cells was significantly reduced at 1 h, just after phagocytosis, in the PG-pretreated macrophages compared with the control macrophages (*P* < 0.01). Phagocytosis at 1 h was not enhanced in the HKLM-pretreated macrophages, whereas phagocytosis of macrophages pretreated with PG + HKLM was significantly enhanced compared with that of macrophages pretreated PG only (*P* < 0.01). On the other hand, almost all intracellular bacteria were low in macrophages of all groups. There was no significant difference the number of intracellular bacteria in macrophages pretreated with PG + HKLM between 1 h and 4 h (*P* > 0.05), because intracellular bacteria were already low.

### 3.6 Effect of PG on uptake of *L. monocytogenes* by macrophages

To further confirm whether decrease of the intracellular number of bacteria is due to decreased uptake by macrophages, lipophilic Dio fluorophore-labelled *L. monocytogenes* cells were used. RAW264.7 cells were pretreated with HKLM in the presence or absence of PG for 24 h and infected with labelled bacteria for 90 min. Non-invasive *L. monocytogenes* was eliminated by 120 μg/mL of gentamicin for 1 h and then the Dio-stained *L. monocytogenes* was observed under a fluorescence microscope (Fig. 7 and Fig. S1). PG pretreatment significantly decreased uptake of *L. monocytogenes* cells in macrophages (*P* < 0.05). In contrast, although uptake of bacterial cells was reduced in HKLM-pretreated macrophages in the presence or absence of PG, the reduction was not statistically significant (*P* > 0.05).

### 3.7 Proteomic analysis of the effect of PG administration of mouse liver

**Fig. 8:**
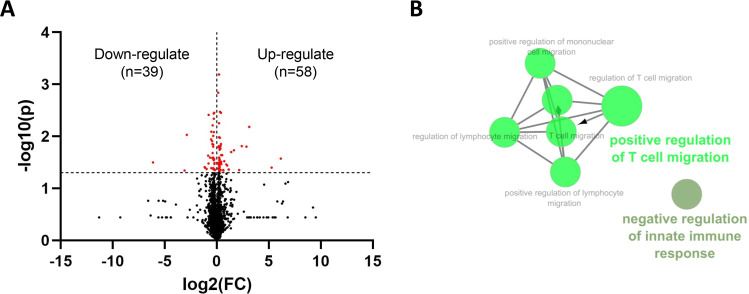
Differential proteomic analysis between liver of PG-treated and control mice. C57BL/6 mice were fed with PG-supplemented diet (3 mg/day) or normal diet from 9 days and the livers were taken for proteomic analysis. (A) Volcano plot of DEPs between PG and NC groups using cutoff -log10(P-value) >1.3 log2FC > 0 or log2FC < 0. Black dots represented proteins without significantly different expression, while the red dots indicated the proteins with significantly differential expression. (B) Functional enrichment of differential expressed genes in PG and NC group analyzed by Cytoscape plugin ClueGO. The enrichment shows only a significant GO term (*P* < 0.05). Node color indicates the specific functional class involved, and bold fonts highlight the most important functional GO terms.

To investigate the differential expression proteins (DEPs) derived from the liver of mice administered with or without PG, proteins were identified by LC-M/MS. DEPs in non-treated control (NC) and PG-treated groups were identified from a total of 2230 proteins, with significant DEPs as shown in Table 1. Using the cut-off values of -log10(P-value) > 1.3 and log2FC > 0 or log2FC < 0 for the analysis of DEPs between the PG-treated and NC groups, the results revealed that 39 proteins were downregulated and 58 proteins were upregulated in the PG group compared to the NC group (Fig. 8). The Cytoscape plugin ClueGO was used to study the function enrichment of differential expression genes (DEGs) from the dataset. The most represented gene ontologies among those 97 components in terms of the immune system are shown in Fig. 8. There were positive regulation of T cell migration (GO:2000,406) and negative regulation of innate immune response (GO:0045,824).

**Table 1:** The down-regulated and up-regulated DEPs in the livers between mice treated with or without PG.

NO.	Genes	Protein	NC*	PG*	P value
Down-regulated					
1	*H6pd*	GDH/6PGL endoplasmic bifunctional protein	6.9	0.1	0.032
2	*Nlgn1; 2; 4l*	Neuroligin 4-like	44.2	5.2	0.046
3	*Lcat*	Phosphatidylcholine-sterol acyltransferase	6.2	0.8	0.009
4	*Hmgcs2*	Hydroxymethylglutaryl-CoA synthase, mitochondrial	446.5	200.3	0.039
5	*Chrac1*	Chromatin accessibility complex protein 1	216.6	99.2	0.039
6	*Ciao1*	Probable cytosolic iron-sulfur protein assembly protein CIAO1	15.3	7.8	0.042
7	*Ppt2*	Lysosomal thioesterase PPT2	49.8	28.2	0.023
8	*Uqcrc1*	Cytochrome b-c1 complex subunit 1, mitochondrial	42.2	24.2	0.033
9	*Gcdh*	Glutaryl-CoA dehydrogenase, mitochondrial	126.5	73.7	0.004
10	*Aco2*	Aconitate hydratase, mitochondrial	369.9	235.1	0.026
11	*Trmt6*	tRNA (adenine(58)-N(1))-methyltransferase non-catalytic subunit TRM6	16.1	11.0	0.011
12	*Rpl34*	60S ribosomal protein L34	254.6	175.9	0.040
13	*Rpl18a*	60S ribosomal protein L18a	27.5	19.1	0.008
14	*Gda*	Guanine deaminase	32.4	23.9	0.012
15	*Sfn*	14–3–3 protein sigma	839.0	620.7	0.026
16	*Ptgr3*	Prostaglandin reductase-3	1858.1	1392.9	0.014
17	*Sec13*	Protein SEC13 homolog	42.4	32.3	0.006
18	*Suclg2*	Succinate–CoA ligase [GDP-forming] subunit beta, mitochondrial	475.9	363.3	0.021
19	*Eif4a2*	Eukaryotic initiation factor 4A-II	212.0	162.3	0.041
20	*Bad*	Bcl2-associated agonist of cell death	35.3	27.8	0.010
21	*Mccc2*	Methylcrotonoyl-CoA carboxylase beta chain, mitochondrial	129.7	102.1	0.026
22	*Nudcd2*	NudC domain-containing protein 2	31.4	24.8	0.015
23	*Ndufs1*	NADH-ubiquinone oxidoreductase 75 kDa subunit, mitochondrial	57.0	45.1	0.016
24	*Aldh9a1*	4-trimethylaminobutyraldehyde dehydrogenase	210.4	167.4	0.026
25	*Cpne1*	Copine-1	78.5	62.6	0.041
26	*Cbr4*	3-oxoacyl-[acyl-carrier-protein] reductase	236.5	189.1	0.040
27	*Glud1*	Glutamate dehydrogenase 1, mitochondrial	970.0	778.7	0.027
28	*Cth*	Cystathionine gamma-lyase	1287.0	1050.0	0.004
29	*Lypla2*	Acyl-protein thioesterase 2	40.5	33.1	0.029
30	*Hsd17b8*	(3R)-3-hydroxyacyl-CoA dehydrogenase	343.4	281.8	0.028
31	*Hebp1*	Heme-binding protein 1	343.7	282.3	0.008
32	*Spryd4*	SPRY domain-containing protein 4	80.5	66.4	0.043
33	*Ubxn1*	UBX domain-containing protein 1	175.2	145.7	0.005
34	*Atp5f1e*	ATP synthase subunit epsilon, mitochondrial	988.1	833.2	0.004
35	*Hspd1*	60 kDa heat shock protein, mitochondrial	1905.7	1610.5	0.028
36	*Ywhag*	14–3–3 protein gamma	294.2	250.1	0.034
37	*Gstm1*	Glutathione S-transferase Mu 1	1069.2	924.5	0.032
38	*Gfus*	GDP-l-fucose synthase	166.7	144.6	0.044
39	*Ivd*	Isovaleryl-CoA dehydrogenase, mitochondrial	69.7	61.6	0.045
40	*Arhgdia*	Rho GDP-dissociation inhibitor 1	1950.0	2152.7	0.032
Up-regulated					
41	*Cat*	Catalase	7650.7	8456.6	0.001
42	*Atxn2*	Ataxin-2	38.5	42.8	0.042
43	*Timm10*	Mitochondrial import inner membrane translocase subunit Tim10	179.4	203.4	0.016
44	*Clip1*	CAP-Gly domain-containing linker protein 1	144.7	165.5	0.034
45	*Tpm1*	Tropomyosin alpha-1 chain	525.5	602.5	0.015
46	*Ubap2l*	Ubiquitin-associated protein 2-like	74.8	85.9	0.036
47	*Znf207*	BUB3-interacting and GLEBS motif-containing protein ZNF207	235.4	274.0	0.001
48	*Eif2s2*	Eukaryotic translation initiation factor 2 subunit 2	117.2	136.5	0.025
49	*Banf1*	Barrier-to-autointegration factor	477.7	559.3	0.049
50	*Lrrfip2*	Leucine-rich repeat flightless-interacting protein 2	60.4	70.9	0.006
51	*Cdc26*	Anaphase-promoting complex subunit CDC26	35.3	41.4	0.010
52	*Slain2*	SLAIN motif-containing protein 2	29.1	34.2	0.035
53	*Mup1*	Major urinary protein 1	65,139.3	76,778.1	0.038
54	*Rab7a*	Ras-related protein Rab-7a	230.7	272.1	0.010
55	*Rhoa*	Transforming protein RhoA	947.6	1119.2	0.046
56	*Pex14*	Peroxisomal membrane protein PEX14	25.0	29.6	0.029
57	*Ctss*	Cathepsin S	839.4	999.2	0.032
58	*Hpcal1*	Hippocalcin-like protein 1	51.8	61.8	0.032
59	*Acin1*	Apoptotic chromatin condensation inducer in the nucleus	111.3	133.1	0.017
60	*Exosc9*	Exosome complex component RRP45	25.3	30.3	0.029
61	*Psmg2*	Proteasome assembly chaperone 2	14.9	17.9	0.040
62	*Septin9*	Septin-9	68.3	82.6	0.027
63	*Washc2*	WASH complex subunit 2	25.6	31.0	0.043
64	*Mup3*	Major urinary protein 3	2655.6	3216.6	0.018
65	*Sf3b1*	Splicing factor 3B subunit 1	61.9	75.0	0.033
66	*Szrd1*	SUZ domain-containing protein 1	125.5	152.3	0.037
67	*Npm1*	Nucleophosmin	976.4	1190.2	0.043
68	*Cbx1*	Chromobox protein homolog 1	157.4	193.4	0.011
69	*Gpalpp1*	GPALPP motifs-containing protein 1	32.7	40.4	0.003
70	*Mup20*	Major urinary protein 20	234.8	290.3	0.016
71	*Canx*	Calnexin	42.4	52.9	0.046
72	*Bsdc1*	BSD domain-containing protein 1	21.2	26.6	0.015
73	*Cap1*	Adenylyl cyclase-associated protein 1	35.6	45.3	0.015
74	*Psap*	Prosaposin	2013.3	2566.3	0.017
75	*Oxsr1*	Serine/threonine-protein kinase OSR1	20.6	26.2	0.034
76	*Mylk*	Myosin light chain kinase, smooth muscle	28.3	36.7	0.045
77	*Tcof1*	Treacle protein	35.6	46.9	0.014
78	*Hp1bp3*	Heterochromatin protein 1-binding protein 3	36.3	48.1	0.004
79	*Pacsin2*	Protein kinase C and casein kinase substrate in neurons protein 2	33.7	45.1	0.031
80	*Ythdf3*	YTH domain-containing family protein 3	34.1	46.1	0.023
81	*Rab14*	Ras-related protein Rab-14	405.7	549.1	0.034
82	*Ddx21*	Nucleolar RNA helicase 2	48.4	69.9	0.023
83	*Mup2*	Major urinary protein 2	32,440.0	50,137.2	0.045
84	*Brk1*	Protein BRICK1	16.6	25.8	0.046
85	*Pnn*	Pinin	19.8	32.6	0.030
86	*Snx5*	Sorting nexin-5	12.1	23.1	0.035
87	*Wnk1*	Serine/threonine-protein kinase WNK1	14.0	28.3	0.037
88	*Ppp1r14a*	Protein phosphatase 1 regulatory subunit 14A	18.8	41.4	0.044
89	*Samhd1*	Deoxynucleoside triphosphate triphosphohydrolase SAMHD1	15.3	34.2	0.044
90	*C6orf132*	Uncharacterized protein C6orf132 homolog	8.6	22.7	0.020
91	*Mcrip1*	Mapk-regulated corepressor-interacting protein 1	8.6	27.2	0.019
92	*Fip1l1*	Pre-mRNA 3′-end-processing factor FIP1	3.2	14.4	0.044
93	*Ddx1*	ATP-dependent RNA helicase DDX1	2.2	11.6	0.016
94	*Krt33a*	Keratin, type I cuticular Ha3-I	3.3	24.2	0.016
95	*Esyt2*	Extended synaptotagmin-2	1.8	15.9	0.007
96	*Polr2a*	DNA-directed RNA polymerase II subunit RPB1	0.1	3.9	0.040
97	*Srprb*	Signal recognition particle receptor subunit beta	0.1	7.2	0.027

⁎ NC: non-treated controls PG: PG-treated samples.

Copine-1 (*P* < 0.05) and UBX domain-containing protein 1 (*P* < 0.01), which repress NF-κB activation pathway (Ramsey et al., 2008; Wang et al., 2015), were down-regulated in the PG-treated mice (Table 1). Meanwhile, leucine-rich repeat flightless-interacting protein 2 was up-regulated in these mice (*P* < 0.01). It is related to NF-κB activation by interaction with TLR4 (Dai, et al., 2009). PG treatment augmented cathepsin S (*P* < 0.05) that is involved in MHC class II-mediated antigen presentation (Baranov et al., 2019). Concerning cytokine response, the inhibitory molecules of type I IFN induction and activity, deoxynucleoside triphosphate triphosphohydrolase SAMHD1 (*P* < 0.05) (Coquel et al., 2018) and YTH domain-containing family protein 3 (*P* < 0.05) (Zhang et al., 2019) were up-regulated in the PG-treated mice. On the other hand, PG treatment augmented Ras-related proteins Rab-7a (*P* < 0.01) and Rab-14 (*P* < 0.05), which are involved in endosome maturation (Seto et al., 2011; Kyei et al., 2006). Moreover, protein kinase C and casein kinase substrate in neurons protein 2 (PACSIN2), which is involved in cell-to-cell spread of *L. monocytogenes*, was up-regulated in the PG-treated mice (*P* < 0.05) (Sanderlin et al., 2019).

## 4 Discussion

We have previously demonstrated that PG suppressed proinflammatory cytokines such as TNF-α and IL-6 but enhanced an anti-inflammatory cytokine, IL-10, induced by heat-killed *Escherichia coli* (HKEC), a Gram-negative bacterium, in mouse macrophage cell line, RAW264.7 cells (Sashinami et al., 2006). In the present study, in contrast, all these cytokines were enhanced by HKLM in RAW264.7 cells as well as BMDM (Fig. 1). In addition, enhanced IL-12 production by HKLM was detected in mDC (Fig. 2). The effect of PG was synergistic in IL-6, IL-10 and IL-12 production, while PG effect was additive in TNF-α production because PG can induce TNF-α production.

It has been known that TNF-α and IFN-γ are critical cytokines in host defense to *L. monocytogenes* infection (Huang et al., 1993; Rothe et al., 1993; Mizuki et al., 2002). IL-6 is also involved in the host defense (Kopf et al., 1994). Furthermore, IL-12 produced early during infection skews naïve CD4^+^ T cells to Th1 cells to establish Th1 cell-dependent host defense to *L. monocytogenes* infection in the adaptive immunity (Pamer, 2004). Therefore, we investigated the effect of PG on host defense to systemic *L. monocytogenes* infection in mice. PG treatment enhanced the elimination of *L. monocytogenes* from the livers and the effective doses of PG were comparable among 1 mg to 5 mg/day (Fig. 3). The number of bacteria in the livers of PG-treated mice was already significantly lower than that of the control mice on day 1 of infection (Fig. 4). These results suggested that daily PG administration augmented host defense to *L. monocytogenes* infection in the stage of innate immunity. The level of serum IFN-γ in the PG-treated mice was significantly higher than that of the non-treated animals on day 1 of infection (Fig. 4). Although PG treatment did not enhance the level of TNF-α in the sera compared with that in non-treated group, enough amounts of TNF-α in the sera were detected. In cytokine response in the spleen cell cultures (Fig. 5), IFN-γ response to HKLM was up-regulated in the PG-treated mice before infection. The TNF-α response was not enhanced by PG treatment, although the response was up-regulated by *L. monocytogenes* infection. These results indicated that up-regulation of IFN-γ production might be important in the effect of PG on host defense to *L. monocytogenes* infection, Moreover, the IL-12p70 response was not enhanced by PG treatment before infection, but the response was markedly augmented in the spleen cells derived from PG-treated mice after infection (Fig. 5) as well as HKLM-stimulated mDC (Fig. 2), suggesting that PG treatment may play a role in Th1 differentiation in *L. monocytogenes* infection.

Some data from proteomic analysis of mouse liver were supported augmentation of host defense to *L. monocytogenes* infection by PG treatment (Table 1 and Fig. 8). NF-κB activation pathway is essential for production of molecules that activate immune response, such as proinflammatory cytokine production. PG treatment up-regulated leucine-rich repeat flightless-interacting protein 2, which is involved in NF-κB activation (Dai, et al., 2009). Conversely, Copine-1 and UBX domain-containing protein 1, which repress NF-κB activation pathway (Ramsey et al., 2008; Wang et al., 2015), were down-regulated in the PG-treated mice. Type I IFN plays protective roles in host defense to viral infections and various bacterial infections (McNab et al., 2015). However, it has been reported that type I IFN impairs host defense to infection with *L. monocytogenes* by apoptosis of T cells, loss of TNF-producing cells, and blocking of the responsiveness of IFN-γ (Auerbuch et al., 2004; Carrero et al., 2004; O’Connell et al., 2004; Rayamajhi et al., 2010). SAMHD1 and YTH domain-containing family protein 3, which are the inhibitory molecules of type I IFN induction and activity (Coquel et al., 2018; Zhang et al., 2019), were up-regulated in the PG-treated mice. Thus, cytokines and cytokine-related molecules for activation of host defense to *L. monocytogenes* might be regulated in liver of the PG-treated mice. On the other hand, IL-10 production induced by HKLM was up-regulated in PG-treated macrophages and mDC (Fig. 1, Fig. 2). IL-10 reportedly plays a detrimental role in host defense to *L. monocytogenes* infection (Liu et al., 2019). However, it is possible that the effect of IL-10 might be covered up by up-regulation of the protective cytokines for host defense to *L. monocytogenes* infection (Figs 1, 2, Fig. 4, Fig. 5).

We have reported that attenuation of systemic inflammation in experimental inflammatory colitis and autoimmune encephalomyelitis mouse models by daily oral administration of PG is associated with a reduction in Th17 lineage differentiation and enhancement of Foxp3^+^ regulatory T cells (Mitsui et al., 2010; Sashinami et al., 2012). In the current study, it was demonstrated that PG can play as an immunostimulatory in *L. monocytogenes* infection. PG augmented proinflammatory cytokines such as TNF-α and IL-6 by HKLM stimulation (Fig. 1, Fig. 2) but suppressed them by HKEC (Sashinami et al., 2006). It has been reported that *L. monocytogenes* is recognized by Toll-like receptor (TLR) 2 and eliminated via TLR2-dependent manner but the defense is independent of TLR4 (Torres et al., 2004; Janot et al., 2008; Moreno-Gonzalo et al., 2017), while lipopolysaccharide from Gram-negative bacteria including *E. coli* reacts via TLR4 (Park et al., 2009; Takeuchi et al., 1999). Therefore, we presumed that PG could induce different responses via TLR2 or TLR4.

*L. monocytogenes* establishes an intracellular niche within macrophages by escaping from phagosomes into the cytosol and spreads cell-to cell (Kaufmann and Dorhoi, 2016). Data from proteomic analysis of mouse liver with PG treatment revealed that Ras-related proteins Rab-7a and Rab-14, which are involved endosome maturation (Seto et al., 2011; Kyei et al., 2006), and PACSIN2, which is involved in cell-to-cell spread of *L. monocytogenes*, were up-regulated (Fig. 8 and Table 1). Therefore, we presumed that the bactericidal activity of macrophages might be enhanced by PG treatment. However, PG treatment diminished uptake of *L. monocytogenes* cells at 1 h into macrophages and the bacteria killing at 4 h was completed irrespective of the treatment (Fig. 6). Uptake of *L. monocytogenes* cells by macrophages using lipophilic Dio fluorophore-labelled bacteria was decreased in the PG-treated RAW264.7 cells (Fig. 7 and Fig. S1). Our previous study indicated that biofilm formation may block uptake of *Pseudomonas aeruginosa* by epithelial cells (Subsomwong et al. 2024). Although *L. monocytogenes* can form biofilm as well as other bacteria (Trang et al., 2023), biofilm formation might not be observed in this experimental condition. It was reported that microRNA-21, which has an anti-inflammatory function, limits uptake of *L. monocytogenes* cells by macrophages (Johnston et al., 2017). A mechanism of the effect of PG herein is currently unclear. Therefore, elucidation by different aspects of possibilities are needed in this action.

## 5 Conclusions

We demonstrated that PG can activate immune responses for host defense against *L. monocytogenes* infection through augmentation of cytokines and limited infection to macrophages. The present study indicates that PG possess quite different potential, namely activation or attenuation of immune responses, depending on the host conditions. This evokes possible applications for prevention of broad diseases.

## Ethic statement

Experiments were approved by the institutional for Animal Care and use Committees (IACUC)/ethics committees of Hirosaki University (Approval no AE01–2022–118, AE01–2023–098, AE01–2024–111).

## CrediT authorship contribution statement

**Akio Nakane:** Writing – original draft, Writing – review and editing, Investigation, Validation, Conceptualization, Funding acquisition, Project administration. **Phawinee Subsomwong:** Writing - review and editing, Investigation, Methodology, Validation, Formal analysis. **Tatsuji Takahashi:** Resources, Validation. **Kenichi Ito**: Resources, Writing - review and editing. **Krisana Asano:** Conceptualization, Validation, Writing – review and editing, Supervision.

## Declaration of competing interest

The authors declare that they have no known competing financial interests or personal relationship that could have appeared to influence the work reported in this paper.

## Data Availability

Data will be made available on request.

## Appendix Supplementary materials

Effect of PG and HKLM on uptake of *L. monocytogenes* by mouse macrophages. Fluorescence microscopy. (*n* = 5/group). Image, image 1
