# Supplementary figures and images for: Salmon nasal cartilage proteoglycan up-regulates Listeria monocytogenes-mediated immune response in mice
Source: Curr Res Microb Sci. 2025 Aug 27;9:100465. doi: 10.1016/j.crmicr.2025.100465 (PMC12446387; doi:10.1016/j.crmicr.2025.100465)

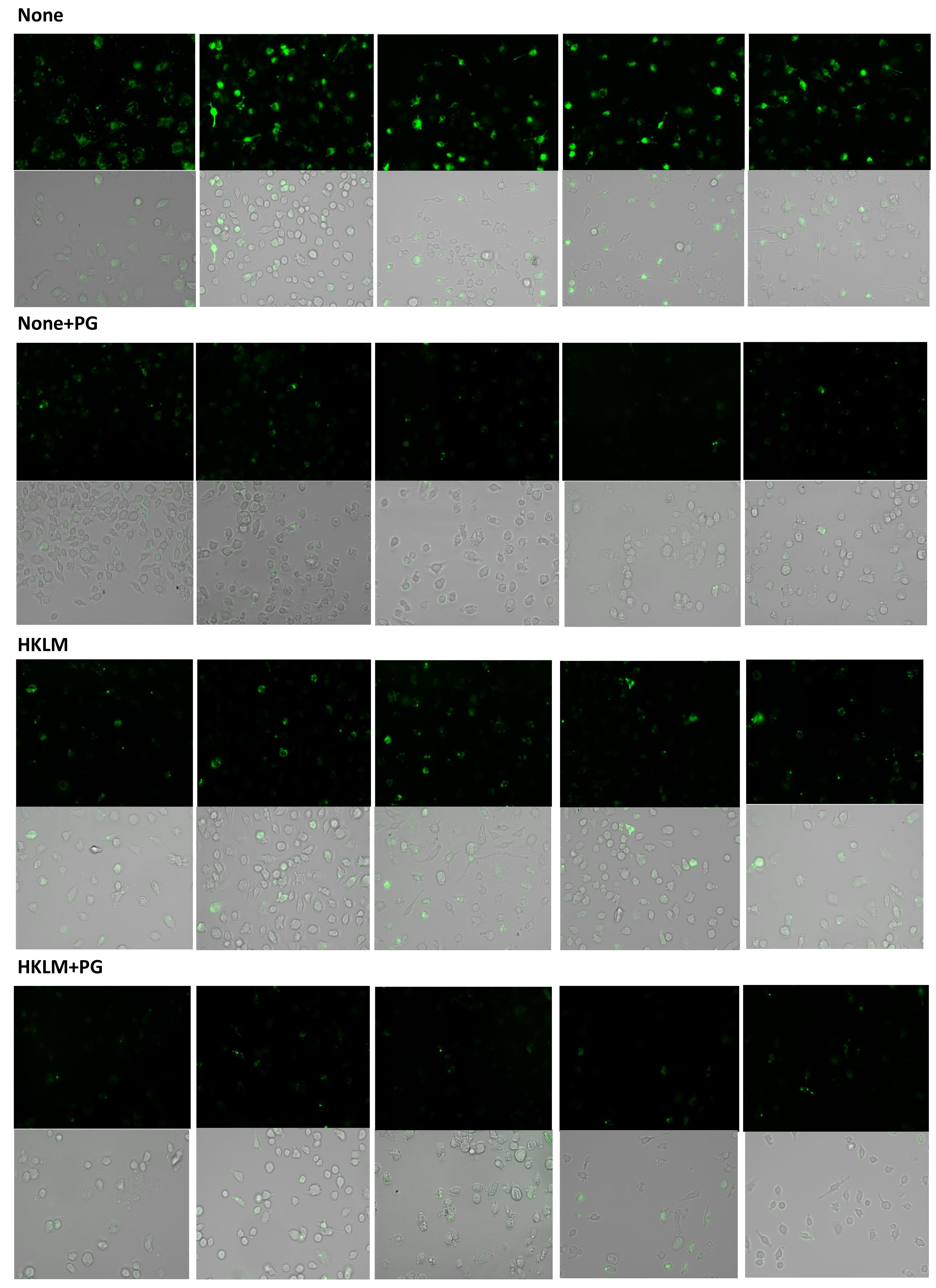

Supplement: Supplementary file 1 — Effect of PG and HKLM on uptake of L. monocytogenes by mouse macrophages. Fluorescence microscopy. (n = 5/group). [file mmc1.jpg]
